# COMBAT-TB-NeoDB: fostering tuberculosis research through integrative analysis using graph database technologies

**DOI:** 10.1093/bioinformatics/btz658

**Published:** 2019-08-26

**Authors:** Thoba Lose, Peter van Heusden, Alan Christoffels

**Affiliations:** South African National Bioinformatics Institute, South African MRC Bioinformatics Unit, University of the Western Cape, Bellville, 7535, South Africa; South African National Bioinformatics Institute, South African MRC Bioinformatics Unit, University of the Western Cape, Bellville, 7535, South Africa; South African National Bioinformatics Institute, South African MRC Bioinformatics Unit, University of the Western Cape, Bellville, 7535, South Africa

## Abstract

**Motivation:**

Recent advancements in genomic technologies have enabled high throughput cost-effective generation of ‘omics’ data from *M.tuberculosis (M.tb)* isolates, which then gets shared via a number of heterogeneous publicly available biological databases. Albeit useful, fragmented curation negatively impacts the researcher’s ability to leverage the data via federated queries.

**Results:**

We present *Combat-TB-NeoDB*, an integrated *M.tb* ‘omics’ knowledge-base. *Combat-TB-NeoDB* is based on Neo4j and was created by binding the labeled property graph model to a suitable ontology namely Chado. *Combat-TB-NeoDB* enables researchers to execute complex federated queries by linking prominent biological databases, and supplementary *M.tb* variants data from published literature.

**Availability and implementation:**

The Combat-TB-NeoDB (https://neodb.sanbi.ac.za) repository and all tools mentioned in this manuscript are freely available at https://github.com/COMBAT-TB.

**Supplementary information:**

Supplementary data are available at *Bioinformatics* online.

## 1 Introduction

Tuberculosis (TB) is a significant global health threat, with one-third of the population infected with its causative agent *Mycobacterium tuberculosis* (*M.tb*). Globally, researchers have been responding with a plethora of heterogeneous TB databases with each focusing on different subsets of TB data and present limited options for data integration thus impeding the chances of integrative analysis. Although each database can provide answers to certain questions in its scope, it falls short in answering questions that require federated queries across multiple domains of biological knowledge.

Mycobrowser (https://mycobrowser.epfl.ch/) remains the preferred curated annotation for *M.tb*. However, information for each genome is presented as a separate application, with no available tools for comparative analysis, and visualization of results. This necessitates a computational platform that can seamlessly integrate a range of data, enabling bioinformaticians to leverage the data via federated *ad hoc* complex queries thus fostering the discovery of new associations between datasets and the validation of existing hypotheses.

Biological data is typically highly connected, semi-structured and relationships are imprecisely known. Graph databases can increase research throughput in the TB research community by bridging the gap between the amount of data produced and the amount of data analyzed. Several biomedical studies have shown that graph databases are well suited for storing and exploring biological models, and offer new insights into analyses (Balaur *et al.*, 2017; Fabregat *et al.*, 2018; Lysenko *et al.*, 2016). Yet the application of graph databases to tuberculosis research remains negligible.

In this work; under the auspices of the Computational Bacterial Analytical Tool kit for TB (COMBAT-TB) (https://combattb.org/) project, we present *Combat-TB-NeoDB*, an integrated *M.tb* ‘omics’ knowledge-base. *Combat-TB-NeoDB* is based on Neo4j (https://neo4j.com/) and enables researchers to execute complex federated queries by linking prominent biological databases, and supplementary TB variants data from published literature or user-generated variants.

## 2 Materials and methods

Researchers have pointed out that the schema-less nature of graph databases can be seen as a double-edged sword in that it provides flexibility, but removes the interoperability standard and necessitates the introduction of consistent semantics by binding the labeled property graph model to a consensus-controlled vocabulary, in the form of a suitable ontology (Lysenko *et al.*, 2016).

### 2.1 Towards a de-facto modeling standard

In an effort to move towards a more de-facto modeling standard we investigated Chado due to its modular and ontology-driven design (Mungall *et al.*, 2007). Relationships between the data are identified using the corresponding Sequence Ontology (SO) terms (Eilbeck *et al.*, 2005).

To model *Combat-TB-NeoDB*, we examined the five core modules that are required by all Chado installations (Supplementary Section S1).

### 2.2 Mapping the Chado schema to Neo4j

Rather than modeling biological entities, or sequence features as an abstract feature and relying on index lookups and JOIN tables to find related data, the labeled property graph model allows for each feature to be modeled as a node in the graph and describes the relationship using the SO terms and or biologically accepted terms (e.g. encodes and translated) (Supplementary Section S1). This provides the ability to retrieve data by traversing relationships.

To store *M.tb* variants, *Combat-TB-NeoDB* uses a variant model inspired by an early version of the GA4GH (https://www.ga4gh.org/) Variants Data Model, with some simplifications (Supplementary Section S1).

### 2.3 Data sources and integration

Prominent biological resources were used to build a database containing the most updated functional genome annotation information with bi-monthly updates (Supplementary Section S2).

To integrate known drug resistance-conferring (DR) variants, we utilized libraries curated by Coll *et al.* (2015) and Feuerriegel *et al.* (2015) due to their extensive systematic review of publicly available databases.

A tool called *tb2neo* (https://github.com/COMBAT-TB/tb2neo) was developed to integrate and import *M.tb* data from the above mentioned biological resources into Neo4j. *tb2neo* takes the H37Rv GFF3 file as input and generates the Combat-TB-NeoDB reference graph database (Supplementary Sections S2 and S3).

A tool called *vcf2neo* (https://github.com/COMBAT-TB/vcf2neo) was developed to takes SnpEff annotated VCF file(s) as input and, using the ANN field, maps these to genes in the ‘reference graph database’ that was imported using the *tb2neo* (Supplementary Section S3).

## 3 Use cases

Neo4j provides several interfaces for multiple programming languages and integrates a browser that can be queried using Cypher, a declarative language*.* Federated queries that are not possible in existing TB portals but possible in *Combat-TB-NeoDB* include extracting known variants from a list of *M.tb* genes, genes that interact with known drug targets, and prioritizing functional variants for further exploration are presented in Supplementary Section S4.

## 4 Discussion

Existing TB data resources are designed and implemented for specific data types. These are either genome, transcriptome or proteome centric and thus fail to answer questions that require federated queries across multiple domains of TB data. A graph database solution, *Combat-TB-NeoDB*, was implemented for researchers to leverage the TB data via federated queries.

Potential queries that can be executed in *Combat-TB-NeoDB* were provided, although the full range of queries is limited only by the user’s biological questions in the context of TB data. As new associations emerge and additional data become available, adding new nodes and relationships to *Combat-TB-NeoDB* without redesigning the schema is trivial. Moreover, Neo4j can be extended using plug-ins to add more complex graph algorithms.

*Combat-TB-NeoDB* forms the backend for our COMBAT-TB Explorer (https://explorer.sanbi.ac.za/) web application.

## Funding

This work was supported by The South African Research Chairs Initiatives of the Department of Science and Technology and National Research Foundation of South Africa grant UID 64751, and the South African Medical Research Council flagship programme MRC-RFA-UFSP-01-2013/ COMBAT-TB.

*Conflict of Interest*: none declared.

## Supplementary Material

btz658_Supplementary_Data
