# Supplementary material for: COMBAT-TB-NeoDB: fostering tuberculosis research through integrative analysis using graph database technologies
Source: Bioinformatics. 2019 Aug 26;36(3):982–3. doi: 10.1093/bioinformatics/btz658 (PMC9883708; doi:10.1093/bioinformatics/btz658)
Supplement: btz658_Supplementary_Data [file bioinformatics_36_3_982_s1.pdf]

Combat-TB-NeoDB Supplementary

Section 1 Towards a de-facto modelling standard

Biological data is typically highly connected, semi-structured and relationships are imprecisely known. Conventionally, biological data is stored in relational database management systems (RDBMS) and flat files. Albeit useful, issues such as transforming the data to conform to a predefined schema, redesigning the schema and application logic on new discoveries, and the computationally expensive use of JOIN queries remain. Considering that one of the fundamental aims in biology is to understand complex relationships among heterogeneous biological data that contribute to biological function, a graph-based approach has been proposed as an alternative to the relational model for storing biological data.
Graph databases can increase research throughput in the TB research community by bridging the gap between the amount of data produced and the amount of data analyzed.

The design of stable, shared schemas that are acceptable to a wide variety of projects is a non-trivial task. In efforts to move towards a more de-facto modelling standard by binding the labelled property graph model to a consensus controlled vocabulary, we investigated Chado, an ontology based RDBMS capable of representing many of the general classes of data encountered in modern biology, and the Sequence Ontology (SO), a structured controlled vocabulary that provides a set of terms and definitions used to facilitate the exchange, analysis and management of genomic data.

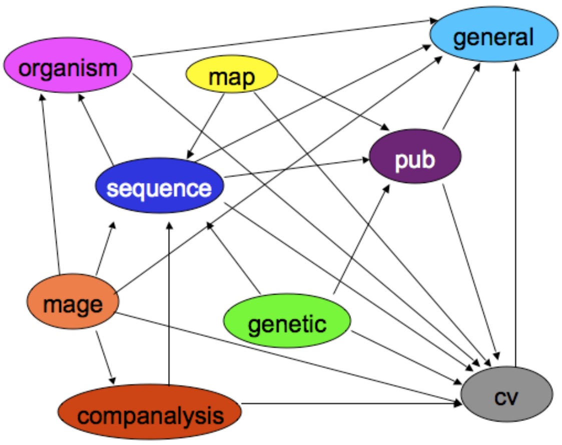

Figure S1: Chado Modules

The Chado schema is built with a set of modules. A Chado module is a set of database tables and relationships that stores information about a well-defined area of biology, such as sequence or attribution. Central to Chado sequence data management is the Sequence module which houses genomic features and things that can be tied to or descend from genomic features.
Diagram obtained from http://gmod.org/wiki/File:ChadoModules.png

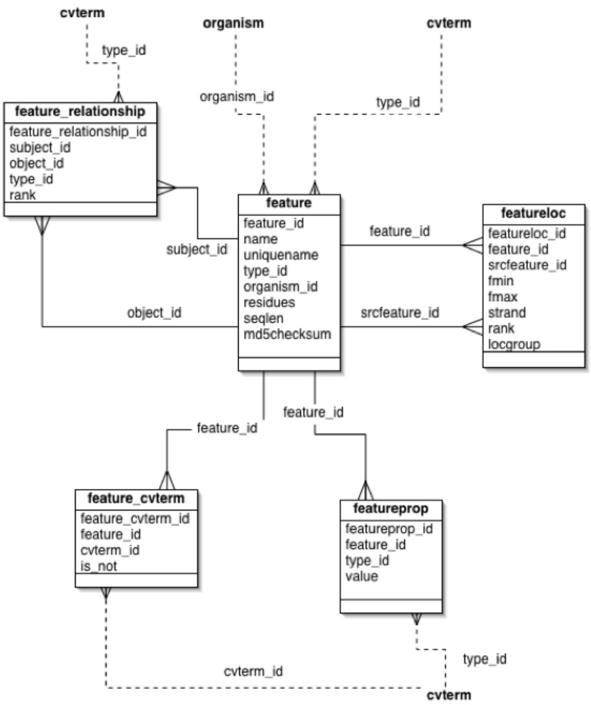

Figure S2: Main tables in Chado Sequence Module

In Chado, all features are stored in the *feature* table, with a very limited set of columns for recording attributes, of the sequence module and feature SO types (gene, transcript, etc.). Relationships are stored, via *feature_relationship* table and *type_id* column, in the *cvterm* table. Columns such a *dbxref_id*and *organism_id* are used to link the feature to its public identifier in the *dbxref* table and the organism it belongs to in the *organism* table. Due to the limited set of columns for recoding attributes, Chado uses the *featureprop* table to store these for any given feature. Figure S2 obtained from http://gmod.org/wiki/File:Feature-tables.png More information can be found on the link below:

- http://www.gmod.org/wiki/Chado_Modules

To model *Combat-TB-NeoDB*, we examined the five core modules that are required by all Chado installations; the Organism, General, Publications, Controlled Vocabulary, and the Sequence module.

Combat-TB-NeoDB Graph Model

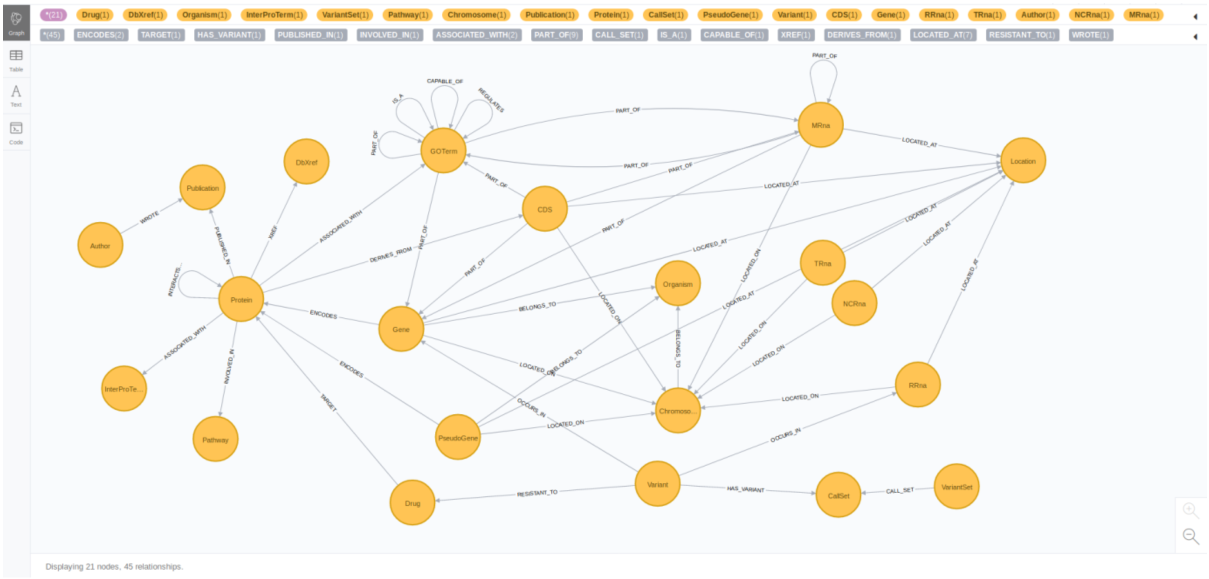

Figure S3: Combat-TB-NeoDB Graph Model

Adapting a RDBMS model to a graph database model is fundamentally a task of converting from ER structure to graph structure. Most notably, a row in a relational model is a node, a table name is represented by a label on nodes, and columns on tables are node properties. Foreign keys and JOIN tables can be used to build edges, thereby transforming loosely coupled data records into a highly bounded group of nodes. The determination and construction of the right relationships is a key activity that impacts the ability of the data structure to respond efficiently to queries.

Rather than modelling biological entities, or sequence features as an abstract feature and relying on index lookups and JOIN tables to find related data, the labelled property graph model allows for each feature to be modelled as a node in the graph and describes the relationship using the SO terms and or biologically accepted terms (e.g. encodes and translated). This provides the ability to retrieve data by traversing relationships.

Variant Data Model

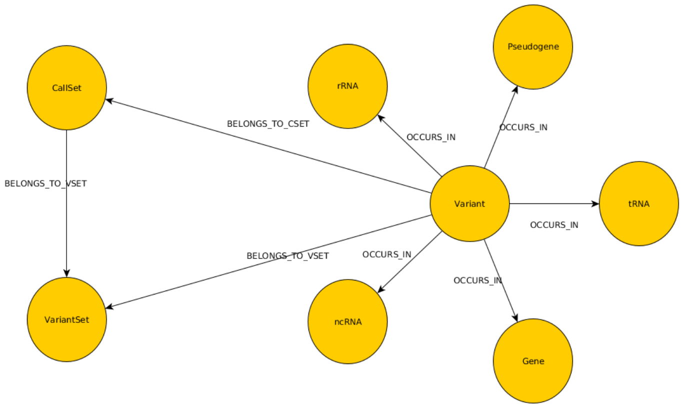

Figure S4:GA4GH inspired Variant Data Model

In our Variant data model, a variant call (Variant) expresses an alternative allele found at a particular genomic location. This location is given in 1-based coordinates relative to the reference sequence (H37Rv). A Variant belongs to a CallSet and multiple CallSets can belong to a VariantSet. This is different to the GA4GH model and expresses the situation where

multiple sets of variant calls are logically associated into a higher level collection. For ease of querying, each Variant is also directly associated with its containing VariantSet. This schema allows rapid search for genomic variants at a particular location. It might, however, change as the discussion on how to model genetic variation in pathogens evolves.
More information about the GA4Gh Variant Data Model can be found in the link below:

- https://ga4gh-schemas.readthedocs.io/en/latest/api/variants.html

Section S2: Data Sources and Integration

Biological Databases

Prominent biological resources were used to build a database containing the most updated functional genome annotation information with bi-monthly updates.

Table S1: A list of public data sources used for data collection

Database	URL
**EnsemblBacteria**	https://bacteria.ensembl.org
**UniProt**	https://www.uniprot.org/
**QuickGo**	https://www.ebi.ac.uk/QuickGO/
**InterPro**	https://www.ebi.ac.uk/interpro/
**KEGG**	https://www.kegg.jp/
**Reactome**	https://reactome.org/
**STRING (v11.0)**	https://string-db.org/
**DrugBank**	https://www.drugbank.ca/
**Pubmed**	https://www.ncbi.nlm.nih.gov/pubmed/

Reference Genome

Annotation of *M.tb* is done primarily using the H37Rv, the most studied strain, as a starting point with additional strains to be added in subsequent releases. A tool called *tb2neo* (https://github.com/COMBAT-TB/tb2neo) was developed to integrate and import *M.tb* data from the above mentioned biological resources into Neo4j. *tb2neo* takes the H37Rv GFF3 file from EnsemblBacteria as input and generates the Combat-TB-NeoDB reference graph database.

TB Variants Libraries

To integrate known drug resistance-conferring variants, we utilised libraries curated by resources in the table below.

Table S2: Variant libraries used

Source	Reference
**TBProfiler**	https://dio.org/10.1186/s13073-015-0164-0
**PhyResSe**	https://dio.org/10.1128/JCM.00025-15

Section S3: Installation

Running Combat-TB-NeoDB locally

To install Combat-TB-NeoDB please follow README file in https://github.com/COMBAT-TB/combat-tb-neodb

Running *tb2neo* locally

To install *tb2neo* please follow README file in https://github.com/COMBAT-TB/tb2neo

Running *vcf2neo* locally

To install *vcf2neo* please follow README file in https://github.com/COMBAT-TB/vcf2neo

Section S4: Use Cases

Querying and analysis on COMBAT-TB NeoDB

Neo4j provides several interfaces for multiple programming languages including Python, a predominant language in bioinformatics, and integrates a web-based, intuitive browser. Integration and exploration of data within the database are done using Cypher, a declarative language.

Using Cypher

Using Cypher, it is possible to perform federated queries in Combat-TB-NeoDB. For example, **finding known variants from a list of genes of interest** (E.g. kasA, and katG).

```
WITH ['kasa','katg'] as genes
MATCH(vs:VariantSet)--(cs:CallSet)--(v:Variant)--(g:Gene)
WHERE tolower(g.name) IN genes
RETURN g.name as gene,  v.consequence as variant,cs.name as variant_collection
```

A second use case could be **finding genes that interact with known drug targets**, considering a score≥0.770, yields 229 genes that interact with known drug targets. (see Table S3 for top 10 results)

```
MATCH(gene:Gene)-[:ENCODES]-(p1:Protein)-[i:INTERACTS_WITH]->(p2:Protein)<-
[:TARGET]-(drug:Drug)
WHERE i.score>= 0.770
RETURN gene.name as Gene, i.score as Score, p2.uniquename as Interactor, drug.name as Drug
ORDER BY Score DESC
```

Another use case could be in *knowledge-driven variant prioritization* where the assessment of genes possessing functional variants in the context of existing biomedical knowledge is vital in producing a manageable set of variants for further exploration. Depending on the study and the biological questions at hand, candidates can be evaluated individually or as a set.

Upon following instructions in https://github.com/COMBAT-TB/vcf2neo and loading SnpEff annotated variants into Combat-TB-NeoDB using *vcf2neo*, **a researcher might want to know if there are any variants in genes that encode known drug targets**.

```
MATCH(vs:VariantSet {name: ‘'MyVariantSet' ’})--(cs:CallSet)--(v:Variant)--(g:Gene)--
(p:Protein)--(d:Drug)
```

```
RETURN g.name as gene, collect(distinct v.consequence) as variants,collect(distinct d.name) as drugs
```

'MyVariantSet' would be the collection/directory containing the user-generated VCF files.

A researcher might also want to **find which variants are associated with pathways** in his/her VariantSet.

```
MATCH(vs:VariantSet {name: ''MyVariantSet' '})--(cs:CallSet)--(v:Variant)--(g:Gene)--(p:Protein)--(pathway:Pathway)
RETURN g.name as gene,collect(distinct v.consequence) as variants,collect(distinct pathway.name) as pathways
```

More example queries can be found here https://combattb.org/combat-tb-neodb.

Table 3: Top 10 results for the Cypher query to find genes that interact with known drug targets

Gene	Score	Interactor	Drug
**rpoA**	0.999	P9WGY7	Rifapentine
**rpoZ**	0.999	P9WGY7	Rifapentine
**folP1**	0.999	P9WNC7	Aminosalicylic Acid
**folB**	0.999	P9WNC7	Aminosalicylic Acid
**thyA**	0.999	P9WNX1	Isoniazid
**fabD**	0.999	P9WNG3	Lauric Acid
**gltB**	0.999	P9WIQ3	Flavin adenine dinucleotide
**pstP**	0.999	P9WI81	Fostamatinib
**atpC**	0.999	P9WPS1	Bedaquiline
**atpD**	0.999	P9WPS1	Bedaquiline

Using Python

The Neo4j community has contributed a range of driver options when it comes to working with the database via Python. These range from lightweight to comprehensive driver packages. See https://neo4j.com/developer/python/ for more information.

We are going to use a python package called *combattbmodel* (https://github.com/COMBAT-TB/combattbmodel). We developed *combattbmodel* to model the NeoDB schema using py2neo (https://py2neo.org/v3/), a client library and toolkit for working with Neo4j from within Python applications and from the command line. This package enables bioinformaticians to interact with Combat-TB-NeoDB using pure Python.

To install *combattbmodel*, run:

```
pip install -i https://test.pypi.org/simple/ combattbmodel
```

The simplest way to try out a connection to Combat-TB-NeoDB is via the console. Once you have started a local Combat-TB-NeoDB instance (https://combattb.org/combat-tb-neodb/installation/), open a new Python console and enter the following code:

Using Python, it is possible to perform federated queries in Combat-TB-NeoDB. For example, **finding known variants from a list of genes of interest** (*katG* and *gyrB*).

```
>>> from py2neo import Graph
>>> graph = Graph(host='localhost', password='')
>>> from combattbmodel.vcfmodel import Variant
>>> genes = ['katG', 'gyrB']
>>> for v in Variant.select(graph):
...     for g in v.occurs_in:
...         if g.name in genes:
...             print(g.name, v.pos, v.consequence)
...
gyrB 6620 Asp461Asn
```

Alternatively:

```
>>> from py2neo import Graph
>>> graph = Graph(host='localhost', password='')
>>> from combattbmodel.vcfmodel import Variant
>>> genes = ['katG', 'gyrB']
>>> for gene in genes:
...     for v in list(Variant.select(graph).where(f"_.gene=~'(?i).*{gene}.*'")):
...         print(g.name, v.pos, v.consequence)
...
katG 2155168 Ser315Thr
```

More example queries can be found here https://combattb.org/combat-tb-neodb.
